# Temporal Trends in Outcomes of ST-Elevation Myocardial Infarction Patients With Heart Failure and Diabetes

**DOI:** 10.3389/fphys.2022.803092

**Published:** 2022-02-03

**Authors:** Bassem Ali, Soha Dargham, Jassim Al Suwaidi, Hani Jneid, Charbel Abi Khalil

**Affiliations:** ^1^Research Department, Weill Cornell Medicine-Qatar, Doha, Qatar; ^2^Heart Hospital, Hamad Medical Corporation, Doha, Qatar; ^3^The Michael E. DeBakey VA Medical Centre, Baylor College of Medicine, Houston, TX, United States; ^4^Joan and Sanford I. Weill Department of Medicine, Weill Cornell Medicine, New York, NY, United States

**Keywords:** heart failure, diabetes, HFREF, HFPEF, STEMI, cardiovascular disease, NIS, mortality

## Abstract

**Aims:**

We aimed to assess temporal trends in outcomes of ST-elevation myocardial infarction (STEMI) patients with diabetes and heart failure with reduced ejection fraction (HFrEF) and heart failure with preserved ejection fraction (HFpEF) and compared both groups.

**Methods:**

Data from the National Inpatient Sample was analyzed between 2005 and 2017. We assessed hospitalizations rate and in-hospital mortality, ventricular tachycardia (VT), ventricular fibrillation (VF), atrial fibrillation (AF), cardiogenic shock (CS), ischemic stroke, acute renal failure (ARF), and revascularization strategy. Socio-economic outcomes consisted of the length of stay (LoS) and total charges/stay.

**Results:**

Hospitalization rate steadily decreased with time in STEMI patients with diabetes and HFrEF. Mean age (SD) decreased from 71 ± 12 to 67 ± 12 (*p* < 0.01), while the prevalence of comorbidities increased. Mortality was stable (around 9%). However, VT, VF, AF, CS, ischemic stroke, and ARF significantly increased with time. In STEMI patients with HFpEF and diabetes, the hospitalization rate significantly increased with time while mean age was stable. The prevalence of comorbidities increased, mortality remained stable (around 4%), but VF, ischemic stroke, and ARF increased with time. Compared to patients with HFrEF, HFpEF patients were 2 years older, more likely to be females, suffered from more cardio-metabolic risk factors, and had a higher prevalence of cardiovascular diseases. However, HFpEF patients were less likely to die [adjusted OR = 0.635 (0.601-0.670)] or develop VT [adjusted OR = 0.749 (0.703-0.797)], VF [adjusted OR = 0.866 (0.798-0.940)], ischemic stroke [adjusted OR = 0.871 [0.776-0.977)], and CS [adjusted OR = 0.549 (0.522-0.577)], but more likely to develop AF [adjusted OR = 1.121 (1.078-1.166)]. HFpEF patients were more likely to get PCI but less likely to get thrombolysis or CABG. Total charges per stay increased by at least 2-fold in both groups. There was a slight temporal reduction over the study period in the LoS of the HFpEF.

**Conclusion:**

While hospitalizations for STEMI in patients with diabetes and HFpEF followed an upward trend, we observed a temporal decrease in those with HFrEF. Mortality was unchanged in both HF groups despite the temporal increase in risk factors. Nevertheless, HFpEF patients had lower in-hospital mortality and cardiovascular events, except for AF.

## Introduction

Heart failure (HF) has been described as a growing pandemic with a significant economic burden. It is estimated that 2.4% of the population currently suffers from HF, which is expected to rise to 3.0% in 2030 (Heidenreich et al., 2013), coupled with an increase in over 100% of the total cost reaching 69.8 billion USD (Heidenreich et al., 2013).

HF is associated with increased morbidity and mortality, especially in the elderly, who are subject to frequent re-hospitalizations (Thrainsdottir et al., 2005; Roth et al., 2015). HF and myocardial infarction (MI) are a common and hazardous combination, with ischemic heart disease remaining the most common cause of HF and a common consequence of it (Torabi et al., 2014; Cahill and Kharbanda, 2017). In a study that examined the association between HF and mortality in patients discharged after their first MI, the 1-year mortality rate was 13.9% in patients with HF compared to 2.4% in patients with no HF (Dunlay et al., 2019). Another study found that up to 10% of patients presenting with the acute coronary syndrome (ACS) have underlying heart failure, which predisposed them to higher in-hospital mortality (Jeger et al., 2017).

Diabetes is associated with higher cardiovascular events (Huang et al., 2017). Patients with HF, MI, or both often encounter diabetes, as they share similar cardio-metabolic risk factors. In adult diabetic patients, the prevalence of HF is estimated to be 9–22%, which is almost 3–4 times the prevalence in the general population (Kaul et al., 2013). On the other side, the prevalence of diabetes in HF patients ranges from 10 to 47%, according to the age and underlying comorbidities (Nichols et al., 2004). Further, HF patients with diabetes have worse clinical outcomes than their non-diabetic counterparts (Allen et al., 2013). Furthermore, diabetes is an independent risk factor for death and re-hospitalizations (Einarson et al., 2018). An improvement in the prevalence, incidence, and outcome of CVD has been noted in the past decades in the general population (Pocock et al., 2013) and diabetes individuals (Abi Khalil et al., 2012). This was fueled by the emergence of new treatments and the comprehensive implementation of prevention guidelines. However, this gradual progress was counteracted by a continuous rise in the costs of the CVD care (Pocock et al., 2013). We, therefore, assessed the temporal trend in cardiovascular and economic outcomes of patients with heart failure with reduced ejection fraction (HfrEF) and with preserved ejection fraction (HFpEF), hospitalized for ST-elevation myocardial infarction (STEMI), and compared both HF entities.

## Materials and Methods

### Data Source

Data were extracted from the national inpatient sample (NIS) database between the years 2005–2017. The database represents almost 20% of de-identified inpatient hospitalizations in the US and about 95% after weighting. The NIS contains clinical and economic data elements related to patients’ demographics, diagnosis, and comorbidities, coded using the International Classification of Disease—9th edition (up till 2014) and ICD-10th edition afterward. The study received administrative IRB approval as it contains only de-identified data (record number 18-00017).

### Diagnosis and Outcomes

The primary diagnosis for this study was STEMI in patients known to have HF and diabetes at inclusion. HF patients were divided into HFrEF and HFpEF based on ICD-9 and ICD-10 used and validated in heart failure studies from the NIS database (Goyal et al., 2018; Lemor et al., 2018; see Appendix). We first assessed temporal trends in baseline characteristics and in-hospital cardiovascular and socio-economic outcomes of STEMI patients with diabetes and either HFrEF or HFpEF between 2005 and 2017. Then, we combined all HFrEF patients and compared them to HFpEF patients for the same outcomes during the observation period. Cardiovascular outcomes included hospitalization rate per 100,000 adults and in-hospital mortality, ventricular tachycardia (VT), ventricular fibrillation (VF), atrial fibrillation (AF), ischemic stroke, acute renal failure (ARF), and cardiogenic shock. The revascularization strategy included percutaneous coronary intervention (PCI), thrombolysis, and coronary artery bypass grafts (CABG). Socio-economic outcomes included length of stay (LoS) and total charges per stay.

### Statistical Analysis

Data for categorical variables are presented using frequency distributions and cross-tabulations and means (standard deviation) and medians (with interquartile range) for continuous variables. Data weighting was used to allow for representative nationwide population estimates as recommended by the Healthcare Cost and Utilization Project, to which the NIS belongs (AHRQ, 2021). Patient-level discharge trend weights consisted of applying the DISCWT variable before 2012 and the TRENDWT variable from 2012 to 2017. Temporal changes were assessed using Trends were analyzed using generalized linear models. Hospitalization costs were adjusted for inflation using numbers provided by the United States Bureau of labor statistics. Comparison of HFrEF with HFpEF patients was performed using a Student’s *t*-test for continuous data and a χ^2^-test for categorical data. Multivariable logistic regression analysis was performed to assess predictors of mortality in both groups. Cardiovascular events were adjusted for baseline characteristics and comorbidities that were statistically different between groups, including age, gender, race, obesity, hypertension, smoking dyslipidemia, peripheral vascular disease, renal failure, and coronary artery disease. We also calculated the Elixhauser comorbidity score, which measures patients’ comorbidities. Initially developed in 1998 by Elixhauser et al. (1998), the score is based on 31 variants and assesses the association of comorbidity with death and future cardiovascular events. Statistical analyses were performed using SPSS (IBM, version 26).

## Results

### Population

**FIGURE 1:**
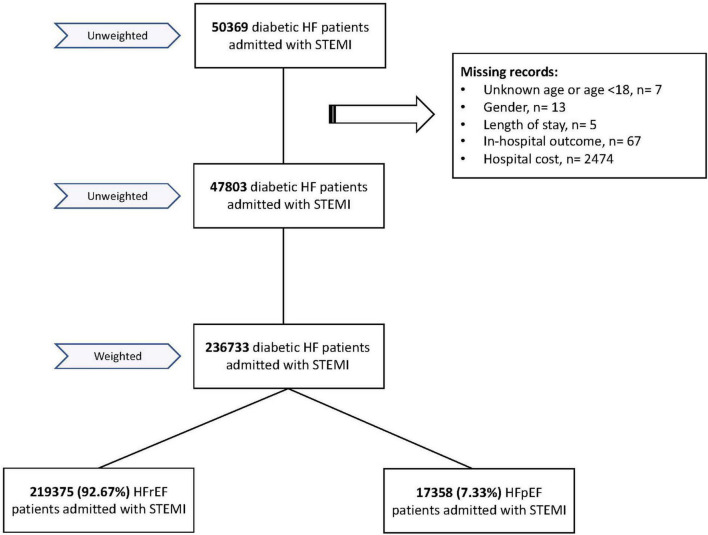
Flow chart of the study.

A total of 47,803 diabetic HF patients admitted for STEMI between 2005 and 2017 were included in our analysis after excluding patients with missing or incomplete records (Figure 1). After weighing the data, our patient population consisted of 236,733 HF patients. Interestingly, most HF patients (92.67%) had HFrEF patients, while only 7.33% had HFpEF.

### Temporal Trend in Characteristics and Outcomes of Heart Failure With Reduced Ejection Fraction Patients

**FIGURE 2:**
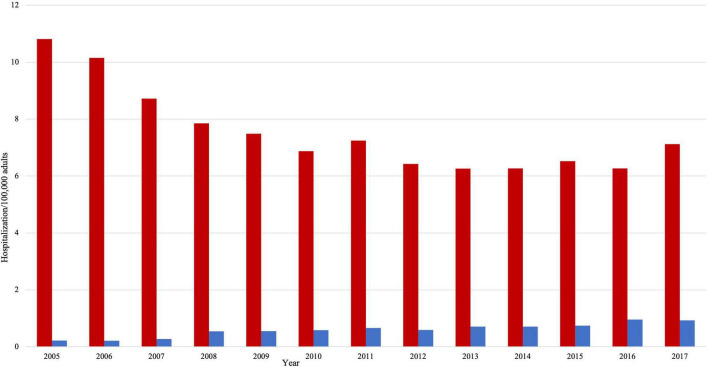
Hospitalizations per 100,000 adults in HFrEF patients (red color) and HFpEF patients (blue color).

Hospitalization rate for HFrEF decreased from 10.81/100,000 adults to 7.12/100,000 adults (Figure 2, *p* trend < 0.001). Over the study period, the mean age (SD) in the HFrEF group decreased from 71.8 (12.5) to 67 (12.6) years old (Table 1, *p* trend < 0.001). The age distribution in the HFrEF group showed significant changes over time: The percentage of patients in the age intervals 75–84 and > 85 gradually decreased, whereas those in the age intervals < 55, 55–64 gradually increased (*p* trend < 0.001 for all). By 2017, 30% of the patients were older than 75 years of age compared to 47% in 2005. The racial distribution changed as well over time as the percentage of white patients decreased from 75.60 to 66.80% (*p* < 0.001), while the percentage of Blacks, Hispanics, and Asians slightly but significantly increased (*p* trend < 0.001 for all). The prevalence of cardiometabolic risk factors such as obesity, hypertension, smoking, and dyslipidemia increased over the study period (*p* < 0.001 for all), which was translated into a substantial increase in the mean (SD) of the Elixhauser comorbidity index. A similar trend was observed in renal failure and coronary artery disease (CAD). Age-adjusted mortality was unchanged, neither was the sex distribution. However, ventricular fibrillation, ventricular tachycardia, atrial fibrillation, ischemic stroke, and acute renal failure increased with time (*p* < 0.001 for all). In terms of revascularization, PCI significantly increased by almost 3-fold (*p* < 0.001) at a time when CABG slightly- and non-significantly- decreased.

**TABLE 1:** Baseline characteristics, outcomes, and temporal trend of HFrEF patients with diabetes admitted for STEMI between 2005 and 2017.

Year	2005	2006	2007	2008	2009	2010	2011	2012	2013	2014	2015	2016	2017	P-Trend
**Age**														
Mean age (SD)	71.84 (12.52%)	71.35 (12.86%)	71.12 (12.94%)	70.96 (12.83%)	70.53 (12.99%)	70.14 (13.13%)	69.43 (13.22%)	68.51 (12.94%)	68.38 (12.81%)	67.80 (12.83%)	67.37 (12.75%)	67.13 (12.70%)	67.05 (12.61%)	< 0.001
<55	2,238 (9.90%)	2,564 (11.80%)	2,130 (11.50%)	1,960 (11.50%)	2,078 (12.60%)	1,989 (13.10%)	2,272 (13.90%)	2,210 (15.00%)	2,190 (15.10%)	2,455 (16.70%)	2,500 (16.60%)	2,570 (17.10%)	2,935 (16.80%)	< 0.001
55–64	4,136 (18.20%)	3,985 (18.40%)	3,678 (19.90%)	3,249 (19.10%)	3,275 (19.90%)	3,163 (20.90%)	3,749 (23.00%)	3,520 (23.90%)	3,455 (23.80%)	3,585 (24.40%)	3,820 (25.30%)	3,895 (25.90%)	4,380 (25.10%)	< 0.001
65–74	5,670 (25.00%)	5,349 (24.70%)	4,405 (23.80%)	4,512 (26.50%)	4,366 (26.50%)	3,960 (26.20%)	4,116 (25.20%)	3,860 (26.20%)	3,920 (27.00%)	3,795 (25.90%)	4,180 (27.70%)	3,990 (26.50%)	5,010 (28.70%)	0.002
75–84	6,957 (30.70%)	6,263 (28.90%)	5,266 (28.40%)	4,706 (27.60%)	4,145 (25.10%)	3,657 (24.20%)	3700 (22.70%)	3,245 (22.00%)	3,145 (21.60%)	3,145 (21.40%)	2,925 (19.40%)	3,075 (20.40%)	3,550 (20.30%)	< 0.001
>84	3,666 (16.20%)	3,491 (16.10%)	3,049 (16.50%)	2,600 (15.30%)	2,633 (16.00%)	2,366 (15.60%)	2464 (15.10%)	1,915 (13.00%)	1,830 (12.60%)	1,690 (11.50%)	1,680 (11.10%)	1,515 (10.10%)	1,585 (9.10%)	< 0.001
**Gender**														
Male	11,659 (51.40%)	11,217 (51.80%)	9,752 (52.60%)	8,848 (52.00%)	9,005 (54.60%)	8,499 (56.20%)	8978 (55.10%)	8,400 (56.90%)	8,435 (58.00%)	8,760 (59.70%)	9,275 (61.40%)	9,425 (62.60%)	10,920 (62.50%)	< 0.001
Female	11,009 (48.60%)	10,434 (48.20%)	8,776 (47.40%)	8,179 (48.00%)	7,492 (45.40%)	6,636 (43.80%)	7,323 (44.90%)	6,350 (43.10%)	6,105 (42.00%)	5,910 (40.30%)	5,830 (38.60%)	5,620 (37.40%)	6,540 (37.50%)	< 0.001
**Race**														
White	12,573 (75.60%)	12,051 (74.00%)	9,953 (72.00%)	10,550 (74.70%)	10,359 (70.60%)	9,220 (69.10%)	10,128 (69.20%)	9,625 (69.00%)	9,655 (70.00%)	9,670 (69.90%)	9,640 (67.80%)	9,560 (66.50%)	11,170 (66.80%)	< 0.001
Black	1,369 (8.20%)	1,549 (9.50%)	1,393 (10.10%)	1,211 (8.60%)	1,512 (10.30%)	1,392 (10.40%)	1,795 (12.30%)	1,595 (11.40%)	1,390 (10.10%)	1,445 (10.40%)	1,660 (11.70%)	1,710 (11.90%)	1,930 (11.50%)	0.003
Hispanic	1,633 (9.80%)	1,689 (10.40%)	1,490 (10.80%)	1,192 (8.40%)	1,517 (10.30%)	1,546 (11.60%)	1,561 (10.70%)	1,560 (11.20%)	1,650 (12.00%)	1,605 (11.60%)	1,710 (12.00%)	1,845 (12.80%)	2,070 (12.40%)	0.001
Asian	369 (2.20%)	432 (2.70%)	384 (2.80%)	467 (3.30%)	456 (3.10%)	487 (3.70%)	488 (3.30%)	375 (2.70%)	460 (3.30%)	425 (3.10%)	520 (3.70%)	495 (3.40%)	750 (4.50%)	0.004
Native American	98 (0.60%)	111 (0.70%)	136 (1.00%)	171 (1.20%)	120 (0.80%)	170 (1.30%)	59 (0.40%)	185 (1.30%)	80 (0.60%)	90 (0.70%)	125 (0.90%)	115 (0.80%)	95 (0.60%)	0.662
Other minority	588 (3.50%)	448 (2.80%)	470 (3.40%)	539 (3.80%)	715 (4.90%)	527 (3.90%)	611 (4.20%)	605 (4.30%)	550 (4.00%)	595 (4.30%)	555 (3.90%)	645 (4.50%)	695 (4.20%)	0.033
**Comorbidities**														
Obesity	2,288 (10.10%)	2,264 (10.50%)	2,073 (11.20%)	2,573 (15.10%)	2,787 (16.90%)	2,620 (17.30%)	3,172 (19.50%)	3,150 (21.40%)	3,235 (22.20%)	3,395 (23.10%)	3,695 (24.50%)	3,820 (25.40%)	4,490 (25.70%)	< 0.001
Hypertension	14,746 (65.10%)	14,399 (66.50%)	12,844 (69.30%)	12,065 (70.90%)	12,679 (76.90%)	11,677 (77.20%)	12,774 (78.40%)	11,795 (80.00%)	11,740 (80.70%)	11,875 (80.90%)	12,675 (83.90%)	11,520 (76.60%)	11,520 (76.60%)	< 0.001
Smoking	3,113 (13.70%)	3,478 (16.10%)	3,489 (18.80%)	3,392 (19.90%)	4,272 (25.90%)	4251 (28.10%)	4,870 (29.90%)	4,900 (33.20%)	4,900 (33.70%)	5,470 (37.30%)	6,040 (40.00%)	5,970 (39.70%)	7,225 (41.40%)	< 0.001
Dyslipidemia	7,912 (34.90%)	8,117 (37.50%)	7,907 (42.70%)	7,764 (45.60%)	8,962 (54.30%)	8595 (56.80%)	9,666 (59.30%)	9,030 (61.20%)	9,100 (62.60%)	9,650 (65.80%)	10,150 (67.20%)	10405 (69.20%)	12,170 (69.70%)	< 0.001
PVD	2,517 (11.10%)	2,469 (11.40%)	2,464 (13.30%)	2,442 (14.30%)	2,608 (15.80%)	2170 (14.30%)	2,560 (15.70%)	2,385 (16.20%)	1,990 (13.70%)	2,360 (16.10%)	2,220 (14.70%)	2030 (13.50%)	1,810 (10.40%)	0.588
Renal failure	3,940 (17.40%)	5,497 (25.40%)	5,470 (29.50%)	4,682 (27.50%)	5,058 (30.70%)	4846 (32.00%)	5,142 (31.50%)	4,750 (32.20%)	4,580 (31.50%)	4,805 (32.80%)	4,760 (31.50%)	5000 (33.20%)	6,085 (34.90%)	0.001
CAD	14,662 (64.70%)	14,301 (66.10%)	12,770 (68.90%)	12,346 (72.50%)	12,621 (76.50%)	12015 (79.40%)	13,221 (81.10%)	12,005 (81.40%)	11,980 (82.40%)	12,425 (84.70%)	12,915 (85.50%)	13780 (91.60%)	15,715 (90.00%)	< 0.001
Elixhauser comorbidity index	36,518 (7.2593)	4.2675 (7.5304)	4.6763 (7.8733)	4.6712 (7.9058)	5.2804 (8.5712)	5.4112 (8.4923)	6.0003 (9.0120)	5.8506 (8.9929)	5.5151 (8.7784)	5.9824 (9.1458)	6.2877 (9.2685)	9.8288 (6.9981)	10.3547 (7.3043)	< 0.001
**Cardiovascular outcomes**														
Mortality (Age-adjusted)	8.79%	8.54%	9.68%	9.14%	7.26%	8.09%	9.60%	10.85%	9.07%	9.72%	10.94%	9.85%	9.28%	0.099
Mortality (Age-adjusted, male)	7.73%	8.35%	9.43%	8.65%	6.27%	8.72%	8.72%	10.53%	8.65%	9.91%	10.40%	9.26%	8.88%	0.09
Mortality (Age-adjusted, female)	8.24%	8.75%	10.04%	9.89%	8.84%	6.40%	11.04%	11.53%	9.88%	10.03%	10.56%	10.67%	9.98%	0.104
Ventricular tachycardia	1,509 (6.70%)	1,714 (7.90%)	1,528 (8.20%)	1,489 (8.70%)	1464 (8.90%)	1,675 (11.10%)	1,720 (10.60%)	1,625 (11.00%)	1,515 (10.40%)	1,855 (12.60%)	1,880 (12.40%)	1,935 (12.90%)	2,380 (13.60%)	< 0.001
Ventricular fibrillation	655 (2.90%)	714 (3.30%)	782 (4.20%)	696 (4.10%)	690 (4.20%)	830 (5.50%)	859 (5.30%)	915 (6.20%)	1,100 (7.60%)	990 (6.70%)	1,175 (7.80%)	1120 (7.40%)	1225 (7.00%)	< 0.001
Atrial fibrillation	4,723 (20.80%)	4,529 (20.90%)	3,900 (21.00%)	3,519 (20.70%)	3,327 (20.20%)	3,404 (22.50%)	3,881 (23.80%)	3,450 (23.40%)	3,405 (23.40%)	3,610 (24.60%)	3,570 (23.60%)	3,610 (24.00%)	4300 (24.60%)	< 0.001
Cardiogenic chock	2,497 (11.00%)	2,659 (12.30%)	2,680 (14.50%)	2,717 (16.00%)	3,164 (19.20%)	3,219 (21.30%)	3,828 (23.50%)	3,485 (23.60%)	3,660 (25.20%)	3,835 (26.10%)	4,080 (27.00%)	3,960 (26.30%)	4,740 (27.10%)	< 0.001
Ischemic stroke	431 (1.90%)	546 (2.50%)	345 (1.90%)	341 (2.00%)	292 (1.80%)	329 (2.20%)	351 (2.20%)	350 (2.40%)	305 (2.10%)	410 (2.80%)	420 (2.80%)	440 (2.90%)	480 (2.70%)	0.003
Acute renal failure	3,370 (14.90%)	3,881 (17.90%)	3,730 (20.10%)	3,839 (22.50%)	4,548 (27.60%)	4,201 (27.80%)	5,049 (31.00%)	4,705 (31.90%)	4,540 (31.20%)	5,330 (36.30%)	5,455 (36.10%)	5,705 (37.90%)	6,615 (37.90%)	< 0.001
**Revascularization strategies**														
PCI	5,789 (25.5%)	6,382 (29.50%)	6,235 (33.70%)	6,331 (37.20%)	6,848 (41.50%)	6,624 (43.80%)	7,821 (48.00%)	7,695 (52.20%)	8,040 (55.30%)	8,570 (58.40%)	9,145 (60.50%)	10,320 (68.60%)	12,425 (71.20%)	< 0.001
Thrombolysis	374 (1.6%)	428 (2.00%)	283 (1.50%)	278 (1.60%)	261 (1.60%)	185 (1.20%)	211 (1.30%)	265 (1.80%)	235 (1.60%)	265 (1.80%)	185 (1.20%)	280 (1.90%)	275 (1.60%)	0.89
CABG	2,100 (9.3%)	2,228 (10.30%)	2,172 (11.70%)	1,657 (9.70%)	1,817 (11.00%)	1,595 (10.50%)	1,913 (11.70%)	1,445 (9.80%)	1,485 (10.20%)	1,415 (9.60%)	1,370 (9.10%)	1,360 (9.00%)	1,455 (8.30%)	0.061

### Temporal Trend in Characteristics and Outcomes of Heart Failure With Preserved Ejection Fraction Patients

The hospitalization rate in HFpEF increased by almost 4 folds, from 0.22/100,000 adults to 0.93/100,000 adults (*p* trend < 0.001). However, there was no statistically significant change in the temporal trend of age and gender (Table 2). By 2017, 38% of the patients were older than 75 years of age compared to 40% in 2005. In a pattern similar to HFrEF patients, cardiovascular risk factors and comorbidities significantly increased with time. For instance, smoking prevalence increased by more than fourfolds (*p* < 0.001). There were no significant changes in age-adjusted mortality and sex distribution. Only ventricular fibrillation and acute renal failure significantly increased among other cardiovascular outcomes (*p* < 0.01 for both). There was a twofold increase in the likelihood of having a PCI during that time (*p* trend < 0.001), but the rates of thrombolysis and CABG were unchanged.

**TABLE 2:** Baseline characteristics, outcomes and temporal trend of HFpEF patients with diabetes admitted for STEMI between 2005–2017.

Year	2005	2006	2007	2008	2009	2010	2011	2012	2013	2014	2015	2016	2017	P-Trend
**Age**														
Mean age (SD)	70.65 (13.161)	71.35 (13.096)	73.71 (11.103)	72.98 (12.352)	72.28 (12.724)	73.46 (12.729)	72.69 (12.246)	70.81 (12.885)	71.77 (12.259)	72.25 (12.073)	70.45 (13.017)	70.02 (12.532)	69.90 (12.794)	0.084
<55	54 (11.90%)	54 (12.00%)	30 (5.30%)	96 (8.30%)	121 (10.00%)	106 (8.50%)	127 (8.70%)	125 (9.30%)	130 (8.10%)	135 (8.30%)	220 (12.90%)	310 (13.60%)	315 (13.90%)	0.184
55–64	79 (17.40%)	106 (23.50%)	104 (18.50%)	195 (16.90%)	230 (19.10%)	217 (17.40%)	254 (17.40%)	295 (21.90%)	330 (20.60%)	270 (16.60%)	330 (19.40%)	470 (20.60%)	425 (18.80%)	0.891
65–74	139 (30.70%)	68 (15.10%)	123 (21.90%)	281 (24.30%)	303 (25.10%)	325 (26.10%)	349 (23.90%)	360 (26.80%)	435 (27.20%)	445 (27.40%)	470 (27.60%)	615 (26.90%)	660 (29.20%)	0.097
75–84	109 (24.10%)	167 (37.00%)	208 (37.10%)	398 (34.40%)	322 (26.70%)	310 (24.90%)	448 (30.60%)	350 (26.00%)	370 (23.10%)	510 (31.40%)	355 (20.80%)	530 (23.20%)	505 (22.30%)	0.028
>84	72 (15.90%)	56 (12.40%)	96 (17.10%)	187 (16.20%)	231 (19.10%)	289 (23.20%)	285 (19.50%)	215 (16.00%)	335 (20.90%)	265 (16.30%)	330 (19.40%)	360 (15.80%)	355 (15.70%)	0.589
**Gender**														
Male	224 (49.40%)	191 (42.40%)	290 (51.60%)	534 (46.20%)	614 (50.90%)	561 (45.00%)	661 (45.20%)	610 (45.40%)	725 (45.30%)	815 (50.20%)	810 (47.50%)	1,045 (45.70%)	1,110 (49.10%)	0.99
Female	229 (50.60%)	259 (57.60%)	272 (48.40%)	623 (53.80%)	593 (49.10%)	686 (55.00%)	802 (54.80%)	735 (54.60%)	875 (54.70%)	810 (49.80%)	895 (52.50%)	1,240 (54.30%)	1,150 (50.90%)	0.99
**Race**														
White	244 (72.80%)	197 (61.90%)	308 (77.80%)	764 (74.30%)	766 (74.80%)	748 (66.30%)	920 (68.70%)	955 (74.60%)	1,020 (66.90%)	1,105 (72.00%)	1,105 (68.60%)	1,505 (68.70%)	1,510 (69.90%)	0.577
Black	64 (19.10%)	68 (21.40%)	44 (11.10%)	112 (10.90%)	104 (10.20%)	160 (14.20%)	208 (15.50%)	125 (9.80%)	200 (13.10%)	165 (10.70%)	225 (14.00%)	255 (11.60%)	275 (12.70%)	0.116
Hispanic	17 (5.10%)	28 (8.80%)	35 (8.80%)	84 (8.20%)	61 (6.00%)	117 (10.40%)	110 (8.20%)	120 (9.40%)	160 (10.50%)	155 (10.10%)	125 (7.80%)	235 (10.70%)	245 (11.30%)	0.015
Asian	0 (0.00%)	10 (3.10%)	9 (2.30%)	25 (2.40%)	26 (2.50%)	52 (4.60%)	47 (3.50%)	25 (2.00%)	65 (4.30%)	45 (2.90%)	60 (3.70%)	90 (4.10%)	85 (3.90%)	0.02
Native American	5 (1.50%)	0 (0.00%)	0 (0.00%)	0 (0.00%)	21 (2.10%)	20 (1.80%)	5 (0.40%)	5 (0.40%)	15 (1.00%)	5 (0.30%)	20 (1.20%)	5 (0.20%)	25 (1.20%)	0.902
Other	5 (1.50%)	15 (72.80%)	0 (0.00%)	43 (4.20%)	46 (4.50%)	32 (2.80%)	50 (3.70%)	50 (3.90%)	65 (4.30%)	60 (3.90%)	75 (4.70%)	100 (4.60%)	20 (0.90%)	0.519
**Comorbidities**														
Obesity	73 (16.10%)	49 (10.90%)	68 (12.10%)	236 (20.40%)	174 (14.40%)	251 (20.10%)	260 (17.80%)	305 (22.70%)	410 (25.60%)	440 (27.10%)	560 (32.80%)	745 (32.60%)	730 (32.30%)	< 0.001
Hypertension	303 (66.90%)	334 (74.20%)	401 (71.40%)	882 (76.20%)	962 (79.70%)	988 (79.30%)	1,254 (85.80%)	1,170 (87.00%)	1,395 (87.20%)	1,375 (84.60%)	1,540 (90.30%)	1,940 (84.90%)	1,940 (84.90%)	< 0.001
Smoking	43 (9.50%)	62 (13.80%)	63 (11.20%)	177 (15.30%)	291 (24.10%)	281 (22.50%)	342 (23.40%)	430 (32.00%)	460 (28.70%)	490 (30.20%)	610 (35.80%)	810 (35.40%)	960 (42.50%)	< 0.001
Dyslipidemia	182 (40.30%)	169 (37.60%)	212 (37.70%)	465 (40.20%)	681 (56.40%)	735 (58.90%)	877 (59.90%)	800 (59.50%)	1,050 (65.60%)	1,065 (65.50%)	1,255 (73.60%)	1,690 (74.00%)	1,575 (69.70%)	< 0.001
PVD	71 (15.70%)	49 (10.90%)	112 (20.00%)	215 (18.60%)	213 (17.60%)	207 (16.60%)	264 (18.00%)	250 (18.60%)	225 (14.10%)	255 (15.70%)	335 (19.60%)	355 (15.50%)	290 (12.80%)	0.803
Renal failure	92 (20.40%)	138 (30.70%)	215 (38.30%)	409 (35.40%)	460 (38.10%)	480 (38.50%)	553 (37.80%)	570 (42.40%)	640 (40.00%)	685 (42.20%)	715 (41.90%)	1,010 (44.20%)	950 (42.00%)	0.001
CAD	271 (59.80%)	289 (64.20%)	390 (69.50%)	778 (67.20%)	923 (76.50%)	978 (78.40%)	1,122 (76.70%)	1,115 (82.90%)	1,290 (80.60%)	1,240 (76.30%)	1,370 (80.40%)	2,015 (88.20%)	2,030 (89.80%)	< 0.001
Elixhauser comorbidity index	3.6518 (7.2593)	4.2675 (7.5304)	4.6763 (7.8733)	4.6712 (7.9058)	5.2804 (8.5712)	5.4112 (8.4923)	6.0003 (9.0120)	5.8506 (8.9929)	5.5151 (8.7784)	5.9824 (9.1458)	6.2877 (9.2685)	9.8288 (6.9981)	10.3547 (7.3043)	< 0.001
**Cardiovascular outcomes**														
Mortality (Age-adjusted)	4.12%	3.60%	14.18%	6.08%	6.94%	7.64%	11.99%	3.55%	6.15%	8.15%	12.84%	3.27%	4.61%	0.947
Mortality (Age-adjusted, Male)	0.54%	2.65%	3.86%	11.07%	7.26%	10.89%	9.54%	3.13%	6.78%	3.80%	12.74%	3.43%	6.39%	0.415
Mortality (Age-adjusted, female)	4.16%	4.20%	23.96%	2.99%	6.33%	3.93%	16.86%	3.88%	3.54%	13.19%	13.21%	2.52%	2.79%	0.723
Ventricular tachycardia	25 (5.50%)	34 (7.60%)	37 (6.60%)	133 (11.50%)	107 (8.90%)	75 (6.00%)	122 (8.30%)	90 (6.70%)	125 (7.80%)	140 (8.60%)	115 (6.70%)	150 (6.60%)	195 (8.60%)	0.867
Ventricular fibrillation	15 (3.30%)	18 (4.00%)	5 (0.90%)	29 (2.50%)	44 (3.60%)	37 (3.00%)	42 (2.90%)	75 (5.60%)	75 (4.70%)	75 (4.60%)	110 (6.50%)	95 (4.20%)	125 (5.50%)	0.01
Atrial fibrillation	73 (16.10%)	116 (25.80%)	171 (30.40%)	199 (17.20%)	241 (20.00%)	287 (23.00%)	392 (26.80%)	390 (29.00%)	465 (29.10%)	525 (32.30%)	490 (28.70%)	525 (23.00%)	615 (27.20%)	0.092
Cardiogenic chock	29 (6.40%)	39 (8.70%)	48 (8.50%)	150 (13.00%)	146 (12.10%)	171 (13.70%)	199 (13.60%)	180 (13.40%)	255 (15.90%)	260 (16.00%)	255 (15.00%)	255 (11.20%)	310 (13.70%)	0.006
Ischemic stroke	15 (3.30%)	15 (3.30%)	14 (2.50%)	30 (2.60%)	15 (1.20%)	15 (1.20%)	46 (3.10%)	25 (1.90%)	30 (1.90%)	25 (1.50%)	50 (2.90%)	50 (2.20%)	40 (1.80%)	0.173
Acute renal failure	79 (17.40%)	94 (20.90%)	105 (18.70%)	311 (26.90%)	354 (29.30%)	375 (30.10%)	453 (31.00%)	445 (33.10%)	540 (33.80%)	600 (36.90%)	565 (33.10%)	795 (34.80%)	765 (33.80%)	<0.001
**Revascularization strategies**														
PCI	123 (27.20%)	140 (31.10%)	144 (25.70%)	365 (31.50%)	426 (35.30%)	464 (37.20%)	701 (47.90%)	670 (49.80%)	845 (52.80%)	810 (49.80%)	955 (56.00%)	1,335 (58.40%)	1,420 (62.80%)	<0.001
Thrombolysis	5 (1.10%)	16 (3.60%)	0 (0.00%)	24 (2.10%)	0 (0.00%)	21 (1.70%)	17 (1.20%)	15 (1.10%)	25 (1.60%)	5 (0.30%)	20 (1.20%)	30 (1.30%)	35 (1.50%)	0.599
CABG	22 (4.90%)	44 (9.80%)	68 (12.10%)	98 (8.50%)	73 (6.10%)	102 (8.20%)	128 (8.70%)	75 (5.60%)	120 (7.50%)	115 (7.10%)	110 (6.50%)	180 (7.90%)	155 (6.90%)	0.372

### Comparison of Both Heart Failure Categories

As seen in Table 3, HFpEF patients were 2 years older, more likely to be females, Blacks, and less likely to be Hispanic (*p* trend < 0.001). They were more likely to smoke and have cardio-metabolic risk factors, such as obesity, hypertension, and dyslipidemia. Cardiovascular diseases, such as PVD, renal failure, and CAD, were more prevalent in HFpEF. Nevertheless, they were less likely to die [adjusted OR = 0.635 (0.601-0.670)] or develop ventricular tachycardia [adjusted OR = 0.749 (0.798-0.940)], ventricular fibrillation [adjusted OR = 0.866 (0.798-0.940)], cardiogenic shock [adjusted OR = 0.549 (0.522-0.577)] or ischemic stroke [adjusted OR = 0.871 (0.776-0.977)] (Table 4). However, atrial fibrillation was significantly higher in HFpEF patients [adjusted OR = 1.121 (1.078-1.166)]. Significant differences were also observed in the treatment of STEMI between the two groups. HFpEF patients were more likely to get PCI [adjusted OR = 1.106 (1.066-1.147)] but less likely to get thrombolysis or CABG [adjusted OR = 0.720 (0.620-0.836), 0.750 (0.703-0.801); respectively] compared to HFrEF patients.

**TABLE 3:** Comparison of baseline characteristics of HFrEF and HFpEF patients with diabetes admitted for STEMI.

		HFrEF	HFpEF	
**Age**	Mean (SD)	69.53 (12.96)	71.47 (12.614)	<0.001
	<55	13.7%	10.50%	<0.001
	55–64	21.80%	19.00%	<0.001
	65–74	26%	26.40%	<0.001
	75–84	24.50%	26.40%	<0.001
	> 84	13.90%	17.70%	<0.001
**Gender**	Male	56.10%	47.20%	<0.001
	Female	43.90%	52.80%	<0.001
**Race**	White	70.50%	70.20%	<0.321
	Black	10.50%	12.60%	<0.001
	Hispanic	11.10%	9.40%	<0.001
	Asian	3.20%	3.40%	0.176
	Native American	0.80%	0.80%	0.664
	Other minorities	4.00%	3.50%	0.006
**Comorbidities**	Obesity	18.00%	24.80%	<0.001
	Hypertension	72.30%	78.90%	<0.001
	Smoking	28.00%	28.90%	0.006
	Dyslipidemia	54.40%	62.00%	<0.001
	PVD	13.70%	16.40%	<0.001
	Renal failure	29.50%	39.90%	<0.001
	CAD	77.80%	79.60%	<0.001
	Elixhauser comorbidity index	5.85 (8.4)	6.94 (8.4)	<0.001

**TABLE 4:** Comparison of outcomes between patients with diabetes admitted for STEMI, either with HFrEF or with HFpEF.

	HFrEF	HFpEF		
				
	N (%)	N (%)	Adjusted OR	
	Unadjusted OR (95% CI)	Unadjusted OR (95%CI)	(95% CI)	*P*-Value
**In-hospital events**				
Mortality	31,728 (14.50%) OR = 1	1,895 (10.90%) OR = 0.713 (0.679-0.749)	0.635 (0.601-0.670)	<0.001
Ventricular tachycardia	22,290 (10.20%) OR = 1	1,348 (7.80%) OR = 0.732 (0.692-0.776)	0.749 (0.703-0.797)	<0.001
Ventricular fibrillation	11,752 (5.40%) OR = 1	747 (4.30%) OR = 0.78 (0.723-0.842)	0.866 (0.798-0.940)	0.001
Atrial fibrillation	49,229 (22.40%) OR = 1	4,488 (25.90%) OR = 1.222 (1.18-1.267)	1.121 (1.078-1.166)	<0.001
Cardiogenic shock	44,524 (20.30%) OR = 1	2,297 (13.20%) OR = 0.582 (0.557-0.609)	0.549 (0.522-0.577)	<0.001
Ischemic stroke	5,040 (2.30%) OR = 1	370 (2.10%) OR = 0.918 (0.825-1.022)	0.871 (0.776-0.977)	0.019
Acute renal failure	60,967 (27.80%) OR = 1	5,481 (31.60%) OR = 1.212 (1.172-1.253)	0.961 (0.924-1.000)	0.05
**Revascularization strategy**				
PCI	102,224 (46.60%) OR = 1	8,400 (48.40%) OR = 1.074 (1.041-1.107)	1.106 (1.066-1.147)	<0.001
Thrombolysis	3,526 (1.60%) OR = 1	212 (1.20%) OR = 0.745 (0.648-0.857)	0.720 (0.620-0.836)	<0.001
CABG	22,012 (10.00%) OR = 1	1,290 (7.40%) OR = 0.706 (0.666-0.748)	0.750 (0.703-0.801)	<0.001

### Predictors of Mortality

The predictors of mortality in both groups are shown in Table 5. As expected, increasing age is associated with increased mortality risk in both groups. Females were slightly protected compared to males in the HFrEF group [OR = 0.93 (0.904–0.956), *p* < 0.001], but no difference in mortality based on gender was reported in HFpEF. Racial characterization showed significant effects on mortality in both groups. In patients with HFrEF, Blacks and Asians had a slightly lower mortality risk compared to White Americans [OR = 0.946 (0.902-0.993), 0.768 (0.706-0.835); respectively], while in the HFpEF group, mortality was increased by almost 50% in Hispanics [OR = 1.579 (1.320–1.888)]. Surprisingly, comorbidities such as hypertension, smoking, and dyslipidemia were associated with decreased mortality in patients with HFrEF (*p* < 0.001 for all). In contrast, obesity was associated with higher mortality risk [OR = 1.145 (1.1–1.191)]. In HFpEF patients, dyslipidemia was also associated with a significant decrease in mortality [OR = 0.551 (0.493–0.615)]. In both groups, the presence of CAD was associated with almost 50% decrease in mortality risk [OR = 0.641 (0.622–0.662) for HFrEF, OR = 0.498 (0.442–0.561) for HFpEF], while the presence of PVD was associated with increased mortality [OR = 1.131 (1.089–1.174) for HFrEF, OR = 1.161 (1.013-1.331) for HFpEF]. Renal failure was associated with significantly higher mortality risk in patients with HFpEF [OR = 1.564 (1.384-1.766)], but not in HFrEF. As expected, a higher Elixhauser comorbidity score was associated with a higher risk of death in both groups. In terms of revascularization, PCI and CABG reduced mortality by almost 50%, but thrombolysis did not have a statistically significant impact.

**TABLE 5:** Predictors of mortality in both entities of heart failure.

Age		HFrEF		HFpEF	
		OR (95% CI)	*P*-value	OR (95% CI)	*P*-value
	<55	Ref	Ref	Ref	Ref
	55–64	1.387 (1.308-1.472)	<0.001	1.170 (0.885-1.547)	0.27
	65–74	1.673 (1.572-1.78)	<0.001	1.084 (0.821-1.430)	0.57
	75–84	2.28 (2.14-2.429)	<0.001	1.512 (1.146-1.996)	0.004
	> 84	2.974 (2.78-3.181)	<0.001	1.979 (1.486-2.636)	< 0.001
**Gender**	Male	Ref	Ref	Ref	Ref
	Female	0.93 (0.904-0.956)	<0.001	0.940 (0.841-1.051)	0.278
**Race**	White	Ref	Ref	Ref	Ref
	Black	0.946 (0.902-0.993)	0.024	0.962 (0.807-1.146)	0.663
	Hispanic	0.999 (0.954-1.047)	0.982	1.579 (1.320-1.888)	< 0.001
	Asian	0.768 (0.706-0.835)	<0.001	1.273 (0.939-1.725)	0.12
	Native American	1.111 (0.945-1.306)	0.203	1.508 (0.854-2.663)	0.157
	Other minorities	1.047 (0.975-1.123)	0.206	1.198 (0.880-1.631)	0.252
**Obesity**	No	Ref	Ref	Ref	Ref
	Yes	1.145 (1.1-1.191)	<0.001	0.882 (0.757-1.027)	0.106
**HTN**	No	Ref	Ref	Ref	Ref
	Yes	0.876 (0.848-0.904)	<0.001	0.969 (0.836-1.123)	0.676
**Smoking**	No	Ref	Ref	Ref	Ref
	Yes	0.762 (0.736-0.788)	<0.001	0.988 (0.868-1.125)	0.853
**Dyslipidemia**	No	Ref	Ref	Ref	Ref
	Yes	0.637 (0.62-0.656)	<0.001	0.551 (0.493-0.615)	< 0.001
**PVD**	No	Ref	Ref	Ref	Ref
	Yes	1.131 (1.089-1.174)	<0.001	1.161 (1.013-1.331)	0.032
**Renal Failure**	No	Ref	Ref	Ref	Ref
	Yes	1.01 (0.979-1.043)	0.48	1.564 (1.384-1.766)	< 0.001
**CAD**	No	Ref	Ref	Ref	Ref
	Yes	0.641 (0.622-0.662)	<0.001	0.498 (0.442-0.561)	< 0.001
**Elixhauser score**		1.038 (1.032-1.045)	<0.001	1.084	< 0.001
**PCI**	No	Ref	Ref	Ref	Ref
	Yes	0.513 (0.485-0.543)	<0.001	0.424 (0.337-0.534)	0.032
**CABG**	No	Ref	Ref	Ref	Ref
	Yes	0.413 (0.367-0.465)	0.48	0.344 (0.186-0.635)	< 0.001
**Thrombolysis**	No	Ref	Ref	Ref	Ref
	Yes	0.867 (0.696-1.081)	0.226	0.860 (0.305-2.423)	0.775

### Temporal Trend in Socio-Economic Outcomes

**FIGURE 3:**
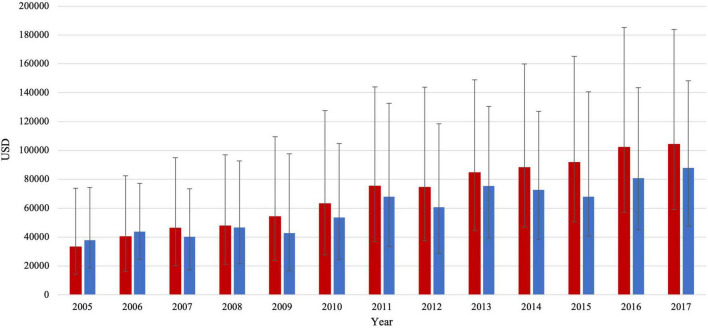
Total charges/stay (median IQR) in HFrEF patients (red color) and HFpEF patients (blue color).

Total charges gradually increased with time in both groups. In patients with HFrEF, total charges per stay increased by almost threefold, from 33,161 (14,193–73,770) to 104,166 (59,052–183,912) USD (adjusted for inflation, *p* trend < 0.001) (Figure 3). In patients with HFpEF, total charges per stay also increased by almost twofold, from 37,892 (18,720–74,254) to 87,972 (47,731–148,151) USD (adjusted for inflation). Of note, total charges were not statistically different between both groups in 2005 but became significantly higher in the HFrEF group over time (*p* < 0.001). In 2005 both groups had a similar median (IQR) LoS of 5 (3–9) days in patients with HFrEF and 6 (3–9) days in patients with HFpEF. There was a slight temporal reduction in the LoS of the HFpEF group, reaching 4 (2–8) days (*p* trend = 0.003). At the same time, no statistically significant changes were observed in the HFrEF group over the study period.

## Discussion

To the best of our knowledge, we report in this analysis that most cases of patients with STEMI with diabetes with pre-existing heart failure are HFrEF. Nevertheless, the percentage of HFpEF significantly increased by almost 4-fold with time while that of HFrEF decreased. Our data is aligned with several other international studies. Tsao et al. (2018) reported a decrease in the incidence rate ratio of HFrEF in the US between 1990 and 2009 while that of HFpEF increased. The Swedish heart failure registry analysis reported similar results between 2000 and 2012 (Chen et al., 2019). The increased recognition of HFpEF as a clinical entity might explain its increasing prevalence, but this explanation is difficult to prove (Oktay et al., 2013). Another possibility could be the temporal increase in cardiovascular risk factors such as diabetes, obesity, and hypertension—primary etiologies of HFpEF—while age-adjusted rates of ischemic heart disease—the most common etiology of HFrEF—are declining in industrialized countries (Dai et al., 2020). Interestingly, in our study that is only focused only on patients with diabetes hospitalized for STEMI, cardiometabolic risk factors significantly increased during the observation period, CAD by 28% in HFrEF and 33% in HFpEF Patients with STEMI and diabetes with pre-existing HFpEF had lower in-hospital mortality than HFrEF patients, concordant with HF patients without STEMI or diabetes studies. In an analysis of 3 multi-national cohorts, lower in-hospital and 2-year mortality was observed in the HFpEF group (Lam et al., 2018). Further, HFpEF patients were older, had a predominance of the female gender, and had a higher prevalence of cardio-metabolic factors than HFrEF, which is aligned with our data.

In our study, the prevalence of men has gradually increased while that of women decreased in HFrEF patients with diabetes. However, no gender-related temporal changes were noted in the HFpEF group. Previous data have demonstrated an apparent gender effect in HF and associated outcomes. Male gender predisposes to HFrEF, probably due to the higher prevalence of the macrovascular coronary disease. In contrast, females tend to develop HFpEF, fueled by coronary microvascular disease and endothelial dysfunction (Lam et al., 2019). There is also an apparent association between gender and mortality in heart failure patients. In our study, the female gender was associated with modest protection against mortality in HFrEF but not in HFpEF. Concordant with our findings, Duca et al. (2018) found that the male gender was associated with increased cardiac mortality in HFrEF. Interestingly, the STAR study reported earlier that females had lower mortality in HFrEF of non-ischemic etiology (Ghali et al., 2003).

Using the NIS database, Ahmed et al. (2014) showed that age-adjusted mortality decreases in diabetic patients hospitalized for acute MI; all categories included. We have recently reported a steady decline in age-adjusted mortality in heart failure and diabetes for the same period (Mekhaimar et al., 2021). Interestingly, mortality is unchanged in our population consisting of diabetic heart failure patients hospitalized for STEMI. One of the plausible reasons is that the increase in the prevalence of risk factors might have counteracted any potential improvement in the outcome of those patients. It is also possible that those patients with three comorbidities (heart failure, diabetes, and STEMI) did not receive the optimal treatment as witnessed by the relatively low revascularization despite its significant increase in recent years when the combination of PCI/thrombolysis was approximately 72% in HFrEF and < 64% in HFpEF.

Contrary to our expectation, several cardio-metabolic risk factors such as hypertension, dyslipidemia, obesity, and strikingly smoking were associated with lower mortality risk. Nevertheless, this paradoxical association has been previously reported in the NIS database in studies assessing the outcome of diabetes patients hospitalized either for MI (Ahmed et al., 2014), heart failure (Mekhaimar et al., 2021), or stroke (Tabbalat et al., 2021). This might be since patients with several risk factors are usually given more cardioprotective medications and have their treatments intensified. Another possible explanation would be the possibility that in most sick patients these cardio-metabolic risk factors are most likely were not accounted for compared to healthier patients with fewer comorbidities, which might give rise to a false impression that these factors are protective.

The continuously growing economic burden of heart failure, STEMI, and diabetes on the healthcare system in the United States is considerable; costs of CVD care are expected to increase by threefold by 2030 (Heidenreich et al., 2011). Further, the simultaneous presence of diabetes in any cardiovascular pathology significantly increases the costs (Nichols and Brown, 2002). We report in this analysis that total charges/stay increased by almost twifold in HFpEF and threefold in HFrEF. Further, total charges/stay were higher in HPrEF patients. This difference aligns with previously reported results in the literature. In a systemic review of the economic costs of heart failure in America, the total charge of hospitalization was 4–9% higher in HFrEF patients than those with HFpEF (Urbich et al., 2020). Another recently published cohort study reported that HFrEF patients had an overall higher economic cost over a 2-year follow-up than those with HFpEF (Vemmos et al., 2012). We anticipate a continuous rise in healthcare spending of both HF types, mainly due to population aging and advances in the medical technologies (Jayawardana et al., 2019).

HFrEF and HFpEF differ in pathophysiology and management. Therefore, it is not surprising that they differ in the risk of some cardiovascular outcomes following hospitalization. While a higher risk of VT (Alvarez et al., 2019), VF (Saour et al., 2017), cardiogenic shock (van Diepen et al., 2017), and ischemic stroke (Murphy et al., 2020) is expected and already known to be associated with a lower LVEF, HFpEF patients had a significantly higher risk of atrial fibrillation. Data in the literature about the risk of AF in STEMI and HF patients is limited. Still, it has been previously reported that AF is generally more prevalent in HFpEF patients than HFrEF. In a study involving more than 40,000 patients with heart failure in the Swedish heart failure registry between 2000 and 2012, Kannel et al. (1983) found that higher ejection fraction correlated with a higher incidence of atrial fibrillation. This might be because HFpEF patients are more obese, knowing that obesity increases the risk of AF by 20–30% (Vyas and Lambiase, 2019).

Several limitations were identified in our study. The retrospective nature of the study design and the absence of randomization limits our ability to reach definitive conclusions. Additionally, several cofounders are missed in the NIS database and could not be considered in our analysis and multivariable regression model. For instance, many strong predictors of mortality in diabetes and heart failure were not available, particularly the left ventricular ejection fraction, glycemic control, and baseline medications. The cause of death in HF patients—and all other patients included in the NIS database—is a weakness in our analysis. Further, our classification of HFpEF and HFrEF was based on systolic and diastolic HF, respectively, as reported in the NIS database. It is not clear what definition was used and whether it was updated with time in the light of newer cardiac guidelines; hence, we cannot exclude the possibility of misclassification between those HF entities in the absence of a LVEF, especially in earlier years when the diagnosis of HFpEF was not well established. Despite these limitations, we believe that our study provided a clear trend in the outcome of diabetic heart failure patients hospitalized for STEMI in a large sample representative of the US population.

## Conclusion

In conclusion, most patients with STEMI with diabetes with pre-existing heart failure are HFrEF patients. While the hospitalization rate of HFpEF patients is steadily increasing, that of HFrEF patients is on a descending slope. Despite the increase in the prevalence of cardiometabolic risk factors in both groups, mortality was unchanged. Finally, HFpEF patients had lower mortality and better cardiovascular outcome except for atrial fibrillation and hemorrhagic stroke. The advances in cardiovascular medicine come at the displayed cost of ongoing medical expenses, which is notably higher in HFrEF patients, even though HFpEF patients were older and had a higher prevalence of CVD and cardiometabolic risk factors.

## Data Availability Statement

The raw data supporting the conclusions of this article will be made available by the authors, without undue reservation.

## Author Contributions

CA conceived the study. BA performed the analysis with SD, CA, JA, and wrote the study. All authors reviewed the final draft and approved it.

## Conflict of Interest

The authors declare that the research was conducted in the absence of any commercial or financial relationships that could be construed as a potential conflict of interest.

## Publisher’s Note

All claims expressed in this article are solely those of the authors and do not necessarily represent those of their affiliated organizations, or those of the publisher, the editors and the reviewers. Any product that may be evaluated in this article, or claim that may be made by its manufacturer, is not guaranteed or endorsed by the publisher.

## Appendix

### Coding of the Diagnosis

#### STEMI

ICD-9 codes: All 410 except 410.7 and its subgroups; ICD-10 codes: I21.0, I21.01, I21.02, I21.09, I21.1, I21.11, I21.19, I21.2, I21.21, I21.29, I21.3, I22.0, I22.1, I22.2, I22.8, I22.9

#### HFrEF

ICD-9 codes: 402.01, 402.11, 402.91, 404.01, 404.03, 404.11, 404.13, 404.91, 404.93, 428.1, 428.20-428.23; ICD-10 codes: I11.0, I13.0, I13.2, I50.1, I50.20-I50.23, I50.40-I50.43, I50.80, I50.81 with its subgroups, I50.82, I50.83, I50.84, I50.89

#### HFpEF

ICD-9 codes: 428.30-428.33; ICD-10 codes: I50.30-I50.33
